# Hsa_circ_0000345 inhibits cell proliferation, migration and invasion of nasopharyngeal carcinoma cells via miR-513a-3p/PTEN axis

**DOI:** 10.1186/s12576-022-00834-4

**Published:** 2022-05-12

**Authors:** Chang Jiang, Hongyan Li, Fei Liu, Linggai Shi, Jun Liu, Yujie Li

**Affiliations:** 1 Department of Otorhinolaryngology, Henan Provincial People’s Hospital (People’s Hospital of Zhengzhou University), No 7 Weiwu Road, Zhengzhou, 450003 China; 2 Department of Otorhinolaryngology, Zhengzhou Central Hospital Affiliated to Zhengzhou University, No 195 Tongbai Road, Zhengzhou, 450007 China

**Keywords:** Hsa_circ_0000345, miR-513a-3p, PTEN, Proliferation, Migration, Invasion, Nasopharyngeal carcinoma

## Abstract

**Background:**

Hsa_circ_0000345 has been reported to be down-regulated in nasopharyngeal carcinoma (NPC). Whether hsa_circ_0000345 can exert antitumor effect in NPC remains unclear. This study aimed to investigate the possible biological role of hsa_cic_0000345 in suppressing the progression of NPC.

**Methods:**

Hsa_circ_0000345 expression was detected in normal nasopharynx epithelial cells (NP69) and NPC cell lines (SUNE1, HONE1, 6-10B and HNE1). The influence of hsa_circ_0000345 on cell proliferation, migration and invasion of NPC cells was evaluated by 3-(4,5-dimethylthiazol-2-yl)-2,5-diphenyltetrazolium bromide (MTT) and transwell assays. Quantitative real-time PCR and western blot were performed to examine gene and protein expression, respectively. Luciferase reporter assay was carried out to verify the relationship among hsa_circ_0000345, miR-513a-3p and phosphatase and tensin homolog deleted on chromosome 10 (PTEN).

**Results:**

Compared with NP69 cells, hsa_circ_0000345 was down-regulated in NPC cells. Moreover, hsa_circ_0000345 overexpression repressed cell proliferation, migration and invasion of SUNE1 cells, whereas hsa_circ_0000345 knockdown promoted cell proliferation, migration and invasion of 6-10B cells. Furthermore, hsa_circ_0000345 promoted PTEN expression by sponging miR-513a-3p. Both miR-513a-3p overexpression and PTEN knockdown promoted cell proliferation, migration and invasion of SUNE1 cells, which were effectively abolished by hsa_circ_0000345 up-regulation.

**Conclusion:**

Hsa_circ_0000345 inhibits cell proliferation, migration and invasion of NPC cells via miR-513a-3p/PTEN axis, thereby suppressing the progression of NPC. Thus, this work suggests that hsa_circ_0000345 may be a potential biomarker for diagnosis and treatment of NPC.

## Background

Nasopharyngeal carcinoma (NPC) is a common malignant tumor that originating from the epithelium of the nasopharyngeal mucosa [1]. NPC is mainly distributed in East Asia and Southeast Asia. According to a rough estimate of the World Health Organization, the number of NPC patients in China accounts for 80% of the global population [2]. The comprehensive treatment of radiotherapy and chemotherapy based on radiotherapy technology has significantly improved the local control rate of NPC. However, due to the insidious onset, early metastasis and easy recurrence of NPC, the 5-year survival rate remains around 70% [3]. Moreover, NPC has the characteristics of high tendency for local metastasis and early distant metastasis, which seriously affects the prognosis of patients. Therefore, analyzing the occurrence and development of NPC from the molecular mechanism level has important clinical significance for improving the survival rate of patients.

Circular RNAs (circRNAs) are a type of covalently closed circular non-coding RNA, which is abundant and stable in various tissues and cells [4]. CircRNAs function as miRNA sponges, RNA binding protein sponges and regulate protein transcription and translation, thus playing an important role in the occurrence and development of various diseases [5]. Previous study has reported that circRNAs are abnormally expressed in NPC and participate in regulating the occurrence and development of NPC [6]. For instance, Yang et al. have found that the promoting effect of curcumin on the sensitivity of NPC to radiotherapy is associated with the abnormal expression of circRNAs. Curcumin significantly promotes the expression of 1042 circRNAs and inhibits the expression of 1558 circRNAs in NPC cells [8]. Hong et al. have confirmed that circCRIM1 is highly expressed in NPC tissues and cells. CircCRIM1 promotes the expression of FOXQ1 by competitively binding miR-422a, thereby promoting the metastasis and chemotherapy resistance of NPC cells [9]. Circ-ZNF609 promotes proliferation, migration and invasion of NPC cells by regulating the miR-188/ELF2 pathway [10]. CircCTDP1 regulates the expression of TGF-β2 through miR-320b/HOXA10 axis, thereby exerting an oncogenic role in NPC [11]. All these findings confirm that circRNAs are closely associated with NPC progression.

Yang et al. have performed high-throughput sequencing analysis on the differentially expressed circRNAs in NPC tissues and normal tissues, finding that 93 circRNAs are up-regulated, and 77 circRNAs are down-regulated in NPC tissues [7]. Among them, hsa_circ_0000345 is significantly down-regulated in the tumor tissues of NPC, suggesting that hsa_circ_0000345 may exert a tumor suppressor effect in NPC. The precise mechanism of action of hsa_circ_0000345 in NPC remains unclear. Recently, it has been reported that phosphatase and tensin homolog deleted on chromosome 10 (PTEN) acts as a target of miR-144-3p, and miR-144-3p accelerates the malignant progression of NPC through directly targeting PTEN [12, 13]. Furthermore, circRNA ITCH promotes PTEN expression by sponging miR-214, and thus repressing the development of NPC [14, 15]. Thus, changes in PTEN expression due to sponging of some miRNAs may promote suppression of NPC development. In the present work, we found that hsa_circ_0000345 released the targeted inhibitory effect of miR-513a-3p on PTEN by competitively binding with miR-513a-3p, thereby inhibiting the proliferation, migration and invasion of NPC cells.

## Materials and methods

### Cell culture

Normal nasopharynx epithelial cells (NP69) and NPC cell lines (SUNE1, HONE1, 6-10B and HNE1) were purchased from Huzhen biotechnology (Shanghai, China). These cells were cultured in RPMI-1640 medium (Gibco, Carlsbad, CA, USA) containing 10% fetal bovine serum (FBS; Gibco) and 1% penicillin/streptomycin (Sangon Biotech, Shanghai, China). Cells were incubated in a constant temperature incubator at 37 °C and 5% CO_2_.

### Plasmid and cell transfection

For overexpression of hsa_circ_0000345, the vector pcDNA3.1 carrying full length of hsa_circ_0000345 was constructed. Empty pcDNA3.1 was used as negative control (NC). To knock down hsa_circ_0000345 or PTEN, small interference RNA (siRNA) specifically targeting hsa_circ_0000345 (Si-hsa_circ_0000345) or PTEN (Si-PTEN), and the corresponding NC (si-NC) were synthesized. The miR-513a-3p mimic, miR-513a-3p inhibitor and the corresponding NC (mimic NC, inhibitor NC) were synthesized. These plasmids were synthesized and purchased from GeneChem (Shanghai, China). Plasmids were transfected into SUNE1 or 6-10B cells using Lipofectamine 2000 Transfection Reagent (Invitrogen, Carlsbad, CA, USA) as the protocol of manufacturer. After 48 h of transfection, the transfected cells were collected for further use. The transfection efficiency of hsa_circ_0000345, miR-513a-3p or PTEN was detected by performing quantitative real-time PCR (qRT-PCR).

### Gene expression

Total RNA was extracted from cells using TRIzol reagent (Invitrogen). The concentration and integrity of RNA was detected using NanoDrop 2000 spectrophotometer (Thermo Fisher Scientific, Waltham, MA, USA) and 1.5% agarose gel electrophoresis. Total RNA was used to synthesize complementary DNA applying SuperScript II first-strand synthesis system (Invitrogen). The relative expression of genes was assessed using TB Green® Premix Ex Taq™ II (Takara, Tokyo, Japan) on ABI Real-time PCR System (Applied Biosystems, Waltham, MA, USA). PCR reaction system (50 μL) contained 25 μL Ex Taq™ II, 2 μL forward primer, 2 μL reverse primer, 4 μL DNA template 1 μL ROX reference dye and 16 μL sterile water. PCR reaction was carried out according to the following conditions: pre-denature at 94 °C for 5 min, followed by 40 cycles of denature at 94 °C for 40 s, anneal at 60 °C for 40 s and extend at 72 °C for 1 min, and finally extend at 72 °C for 10 min. The primers used in qRT-PCR were shown as follows: hsa_circ_0000345: 5’-GTGGCAATTATCCCCAAACTGT-3’ (forward) and 5’-GGTGGAAGAAGAGTCAACAGC-3’ (reverse); miR-513a-3p: 5’-TAAATTTCACCTTTCTGAGAAGG-3’ (forward) and 5’-GCGAGCACAGAATTAATACGAC-3’ (reverse); PTEN: 5’-CGACGGGAAGACAAGTTCAT-3’ (forward) and 5’-AGGTTTCCTCTGGTCCTGGT-3’ (reverse); GAPDH, 5’-CAAGGTCATCCATGACAACTTTG-3’ (forward) and 5’-GTCCACCACCCTGTTGCTGTAG-3’ (reverse); U6: 5’-CGCTTCGGCAGCACATATACTA-3’ (forward) and 5’-CGCTTCACGAATTTGCGTGTCA-3’ (reverse). GAPDH served as loading control for hsa_circ_0000345 and PTEN. U6 served as loading control for miR-513a-3p. The results were analyzed using 2^−∆∆CT^ method for quantification.

### Cell proliferation

Cell proliferation was estimated by performing 3-(4,5-dimethylthiazol-2-yl)-2,5-diphenyltetrazolium bromide (MTT) assay applying MTT Cell Proliferation and Cytotoxicity Assay Kit (Beyotime, Shanghai, China). Briefly, cells were seeded into a 96-well plate at concentration of 2000 cells/100 μL. MTT reagent (10 µL, 5 mg/mL) was added into each well and incubated at 37 °C. After 4 h of incubation, cells were incubated with Formazan at 37 °C for 4 h. The absorbance of samples was detected at 570 nm wavelength using microplate reader (Thermo Fisher Scientific).

### Cell migration and invasion

Transwell assay was performed to estimate cell migration and invasion using 24-well transwell insert system (Corning, NY, USA). For cell migration, 200 μL of cells was seeded into the upper chamber at concentration of 1.5 × 10^6^ cells/mL. The lower chamber supplemented with RPMI-1640 medium and 10% FBS. Cells were incubated in the chamber at 37 °C for 24 h. After that, the un-migrated cells were removed from the upper side of chamber. The migrated cells were stained using 1% crystal violet (Solarbio, Beijing, China) for 5 min.

For cell invasion, the basolateral transwell chambers were covered with 1 mg/mL Matrigel (Becton Dickinson Biosciences, San Diego, CA, USA). Except for this step, cell invasion assay had the same steps with cell migration assay. Finally, images of cell migration and invasion were obtained using an inverted microscope (Olympus, Tokyo, Japan), and then analyzed using Image J software.

### CircRNA–miRNA–mRNA interaction prediction

We used bioinformatics software Circinteractome (https://circinteractome.nia.nih.gov/) and Targetscan (http://www.targetscan.org) to predict the binding of the miRNA and hsa_cic_0000345, and the potential target for miRNA, respectively. The prediction results revealed that there were binding sites between hsa_circ_0000345 and miR-513a-3p, and PTEN may be the down-stream target for miR-513a-3p.

### Luciferase reporter assay

The wild-type (WT) or mutant type (Mut) of hsa_circ_0000345 and PTEN containing the predicted binding sites of miR-513a-3p were cloned into pmir-GLO vector (Promega, Madison, USA), generating the vectors pmir-GLO-hsa_circ_0000345-WT, pmir-GLO-hsa_circ_0000345-Mut, pmir-GLO-PTEN-WT and pmir-GLO-PTEN-Mut (GeneChem). The WT/Mut hsa_circ_0000345 vector, WT/Mut 3' untranslated region (UTR) of PTEN vector and miR-513a-3p mimic or mimic NC were co-transfected into 293 T cells. The relative luciferase activity of cells was detected after 48 h of transfection using Dual luciferase assay kit (Promega).

### Protein expression

Total protein was extracted from cells using Total Protein Extraction Kit (Solarbio) following the protocol described. BCA Protein Assay Kit (Solarbio) was used to detect the concentration of proteins. Protein samples (25 μg) were separated by 10% SDS-PAGE protein electrophoresis, and then transferred onto PVDF membranes (Merck Millipore, Billerica, MA, USA). The membranes were incubated with 5% skimmed milk to block nonspecific sites, and then incubated with the primary antibody, PTEN (1:1000 dilution; Proteintech, Wuhan, China) at 4 °C for 12 h. Horseradish peroxidase-conjugated second antibody (1:5000 dilution; Proteintech) was incubated with the membranes. β-actin antibody (1:5000 dilution; Proteintech) was used as a reference protein for normalization. The data were analyzed by Image J software.

### Statistical analysis

Data were obtained under 3 independent biological reduplications condition, and represented as mean ± standard deviation. SPSS 22.0 statistical software (IBM, Armonk, NY, USA) was used for statistical analysis. Two-tailed Student’s *t* test, one-way ANOVA was used to analyze the statistical difference. *P* < 0.05 was considered as a significant difference.

## Results

### Hsa_circ_0000345 repressed cell proliferation, migration and invasion of NPC cells

**Fig. 1:**
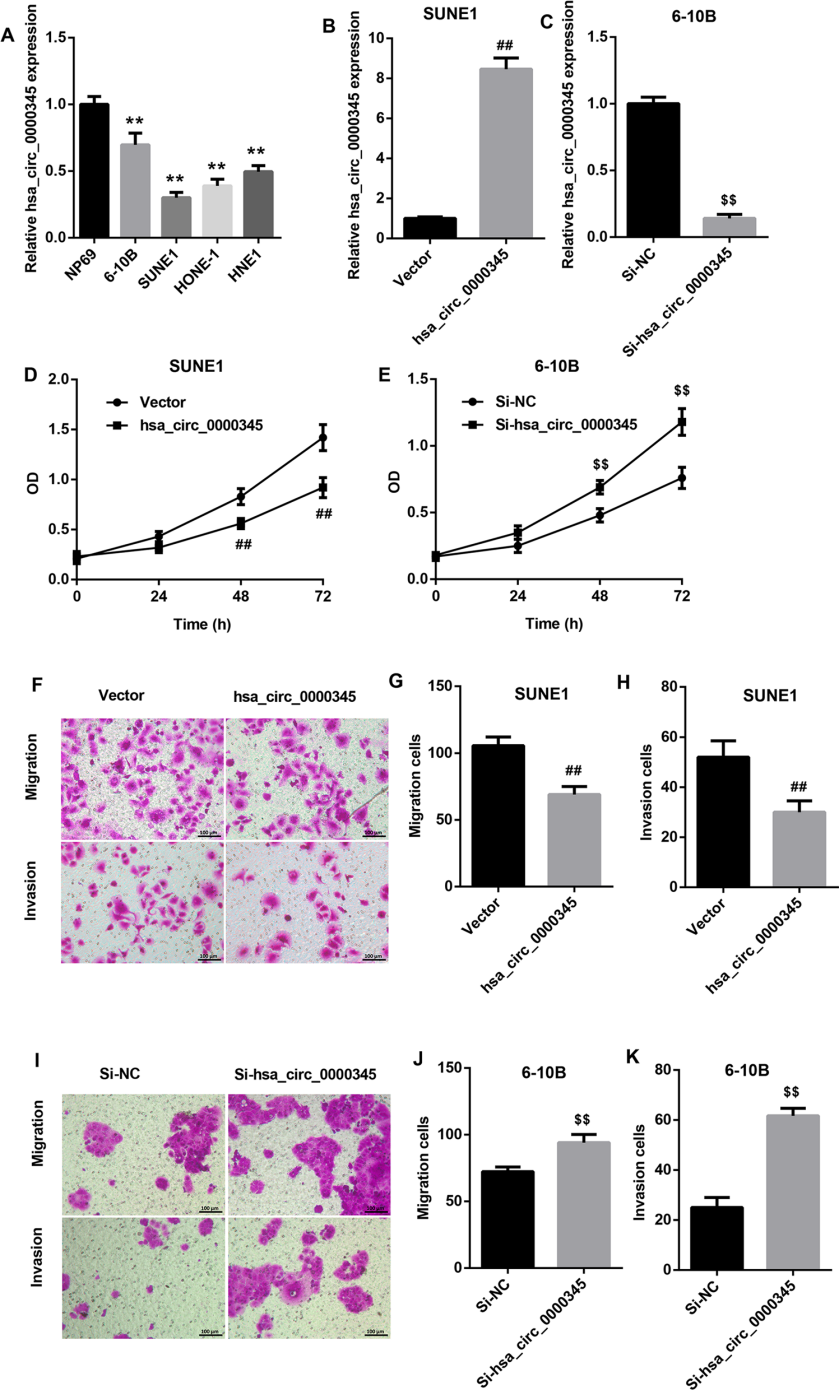
Hsa_circ_0000345 overexpression repressed cell proliferation, migration and invasion of SUNE1 cells, and hsa_circ_0000345 knockdown promoted cell proliferation, migration and invasion of 6-10B cells. **A** QRT-PCR was performed to detect the expression of hsa_circ_0000345 in NP69, SUNE1, HONE1, 6-10B and HNE1 cells. SUNE1 cells were transfected with pcDNA3.1-hsa_circ_0000345 or pcDNA3.1-NC. 6-10B cells were transfected with Si-hsa_circ_0000345 or Si-NC. **B**, **C** QRT-PCR was performed to assess the expression of hsa_circ_0000345 in the transfected SUNE1 and 6-10B cells. **D**, **E** MTT assay was performed to examine cell proliferation of the transfected SUNE1 and 6-10B cells. (F-K) Transwell assay was performed to estimate cell migration and invasion of the transfected SUNE1 and 6-10B cells. **P < 0.01 vs. NP69; ^##^*P* < 0.01 vs. Vector; ^$$^*P* < 0.01 vs. Si-NC

We initially compared the expression of hsa_circ_0000345 between normal nasopharynx epithelial cells and NPC cells. Compared with NP69 cells, hsa_circ_0000345 was down-regulated in SUNE1, HONE-1, 6-10B and HNE1 cells, although at different extents (Fig. 1A). To determine the biological role of hsa_circ_0000345 in NPC, we overexpressed hsa_circ_0000345 in SUNE1 cells that express relatively lower expression of hsa_circ_0000345. Hsa_circ_0000345 was knocked down in 6-10B cells that express relatively higher expression of hsa_circ_0000345. Hsa_circ_0000345 was up-regulated in the SUNE1 cells in presence of pcDNA3.1-hsa_circ_0000345, whereas hsa_circ_0000345 was down-regulated in the 6-10B cells following transfection with Si-hsa_circ_0000345 (Fig. 1B, C). Moreover, the data of MTT and transwell assays revealed that hsa_circ_0000345 overexpression led to a severe decrease in cell proliferation, migration and invasion of SUNE1 cells (Fig. 1D, F, G and H). We also found that hsa_circ_0000345 deficiency significantly promoted cell proliferation, migration and invasion of 6-10B cells (Fig. 1E, I, J and K). All these findings showed that Hsa_circ_0000345 repressed malignant phenotypes of NPC cells.

### Hsa_circ_0000345 promoted PTEN expression by sponging miR-513a-3p

**Fig. 2:**
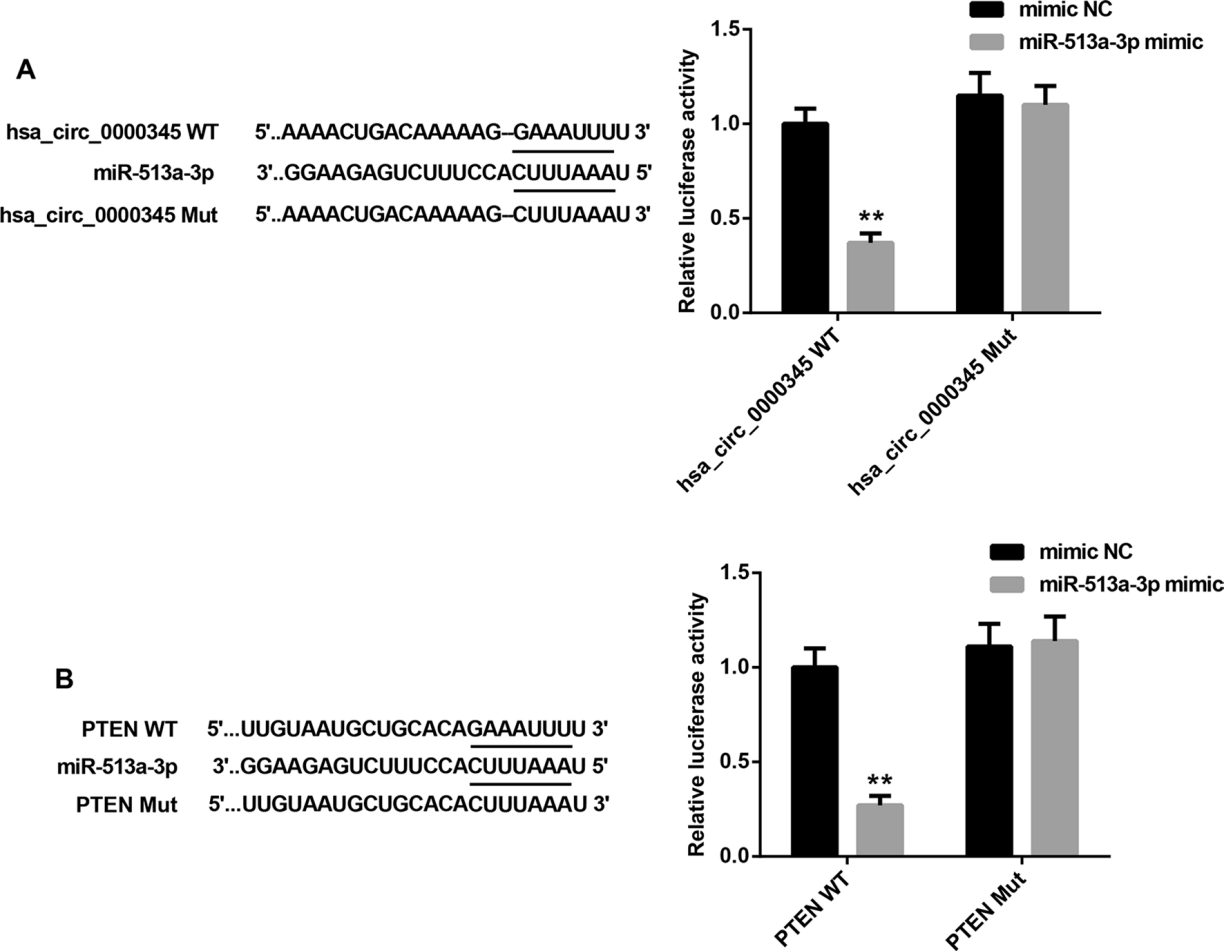
Hsa_circ_0000345 interacted with miR-513a-3p, and miR-513a-3p interacted with PTEN. **A**, **B** Luciferase reporter assay was performed to determine the relationship among hsa_circ_0000345, miR-513a-3p and PTEN in 293 T cells. **P < 0.01 vs. mimic NC

**Fig. 3:**
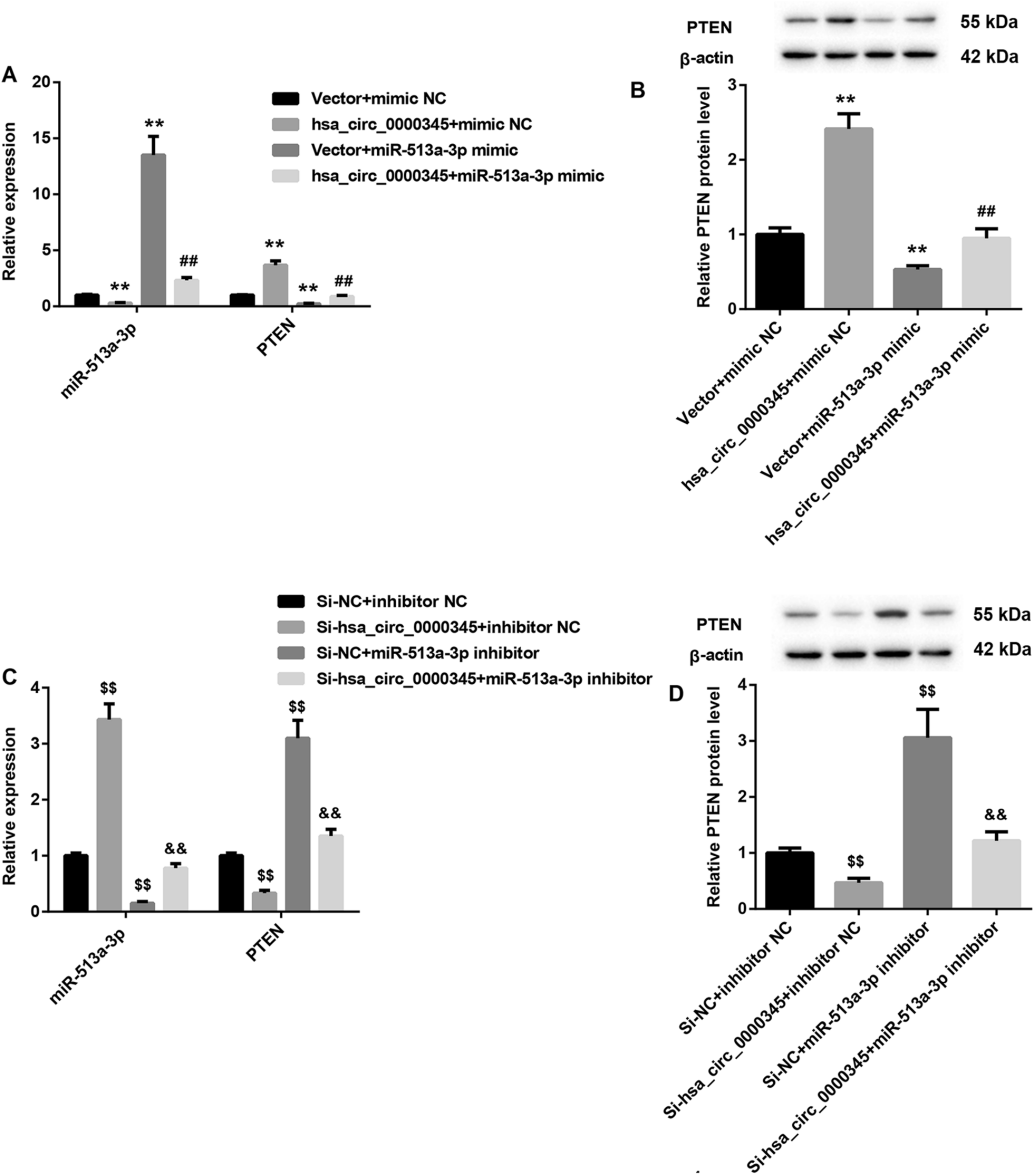
Hsa_circ_0000345 promoted PTEN expression by repressing miR-513a-3p. SUNE1 cells were co-transfected with pcDNA3.1-hsa_circ_0000345 or pcDNA3.1-NC and miR-513a-3p mimic or mimic NC. **A** QRT-PCR was performed to assess the expression of miR-513a-3p and PTEN in the transfected SUNE1 cells. **B** WB was performed to examine the expression of PTEN in the transfected SUNE1 cells. 6-10B cells were transfected with Si-hsa_circ_0000345 or Si-NC and miR-513a-3p inhibitor or inhibitor NC. **C** QRT-PCR was performed to assess the expression of miR-513a-3p and PTEN in the transfected 6-10B cells. **D** WB was performed to examine the expression of PTEN in the transfected 6-10B cells. **P < 0.01 vs. Vector + mimic NC; ^##^*P* < 0.01 vs. Vector + miR-513a-3p mimic; ^$$^*P* < 0.01 vs. Si-NC + inhibitor NC; ^&&^*P* < 0.01 vs. Si-NC + miR-513a-3p inhibitor

Then, we sought to determine the underlying antitumor effect of hsa_circ_0000345 in NPC. We used bioinformatics software Circinteractome to predict the miRNA that hsa_circ_0000345 might bind to. The prediction results showed that there were binding sites between hsa_circ_0000345 and miR-513a-3p. The bioinformatics software Targetscan was used to predict the potential target for miR-513a-3p, showing that miR-513a-3p may interact with PTEN. We performed luciferase reporter assay to verify this prediction. Exactly, miR-513a-3p was a target of hsa_circ_0000345, and there were binding sites between miR-513a-3p and PTEN (Fig. 2A, B). Moreover, hsa_circ_0000345 overexpression significantly repressed miR-513a-3p expression, while miR-513a-3p overexpression caused an up-regulation of miR-513a-3p in SUNE1 cells. Hsa_circ_0000345 overexpression impaired miR-513a-3p overexpression-mediated promotion of miR-513a-3p expression in SUNE1 cells (Fig. 3A). In contrast, hsa_circ_0000345 overexpression enhanced the mRNA and protein levels of PTEN in SUNE1 cells. Overexpression of miR-513a-3p led to a down-regulation of PTEN mRNA and protein in SUNE1 cells, which was effectively rescued by hsa_circ_0000345 overexpression (Fig. 3B). In addition, hsa_circ_0000345 silencing promoted the expression of miR-513a-3p, whereas miR-513a-3p knockdown reduced miR-513a-3p expression in 6-10B cells. The influence conferred by miR-513a-3p knockdown was partly abolished by hsa_circ_0000345 deficiency (Fig. 3C). Furthermore, the mRNA and protein levels of PTEN were inhibited by hsa_circ_0000345 deficiency, and enhanced by miR-513a-3p knockdown in 6-10B cells. Knockdown of hsa_circ_0000345 abolished miR-513a-3p knockdown-mediated promotion of PTEN in 6-10B cells (Fig. 3D). Therefore, hsa_circ_0000345 promoted PTEN expression by sponging miR-513a-3p.

### Hsa_circ_0000345 repressed cell proliferation, migration and invasion of NPC cells by regulating miR-513a-3p/PTEN axis

**Fig. 4:**
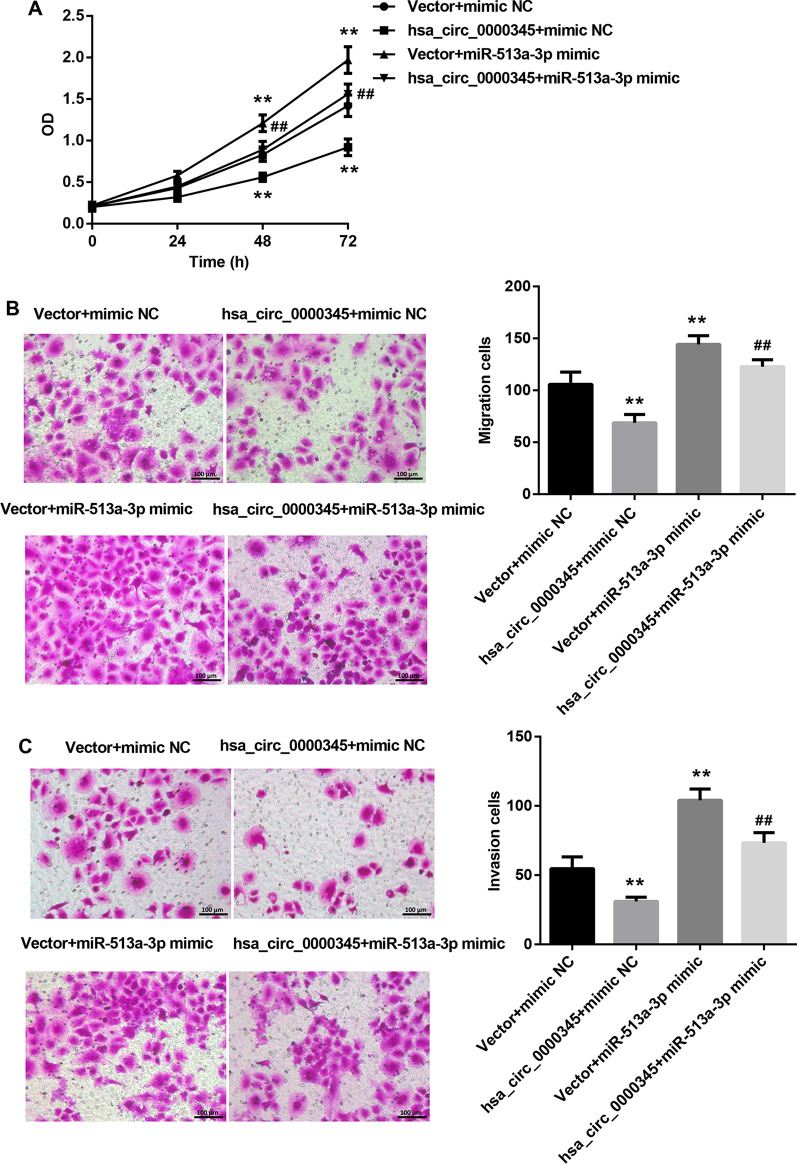
Hsa_circ_0000345 repressed cell proliferation, migration and invasion of SUNE1 cells by repressing miR-513a-3p. SUNE1 cells were co-transfected with pcDNA3.1-hsa_circ_0000345 or pcDNA3.1-NC and miR-513a-3p mimic or mimic NC. **A** MTT assay was performed to examine cell proliferation of the transfected SUNE1 cells. **B**, **C** Transwell assay was performed to estimate cell migration and invasion of the transfected SUNE1 cells. **P < 0.01 vs. Vector + mimic NC; ^##^*P* < 0.01 vs. Vector + miR-513a-3p mimic

**Fig. 5:**
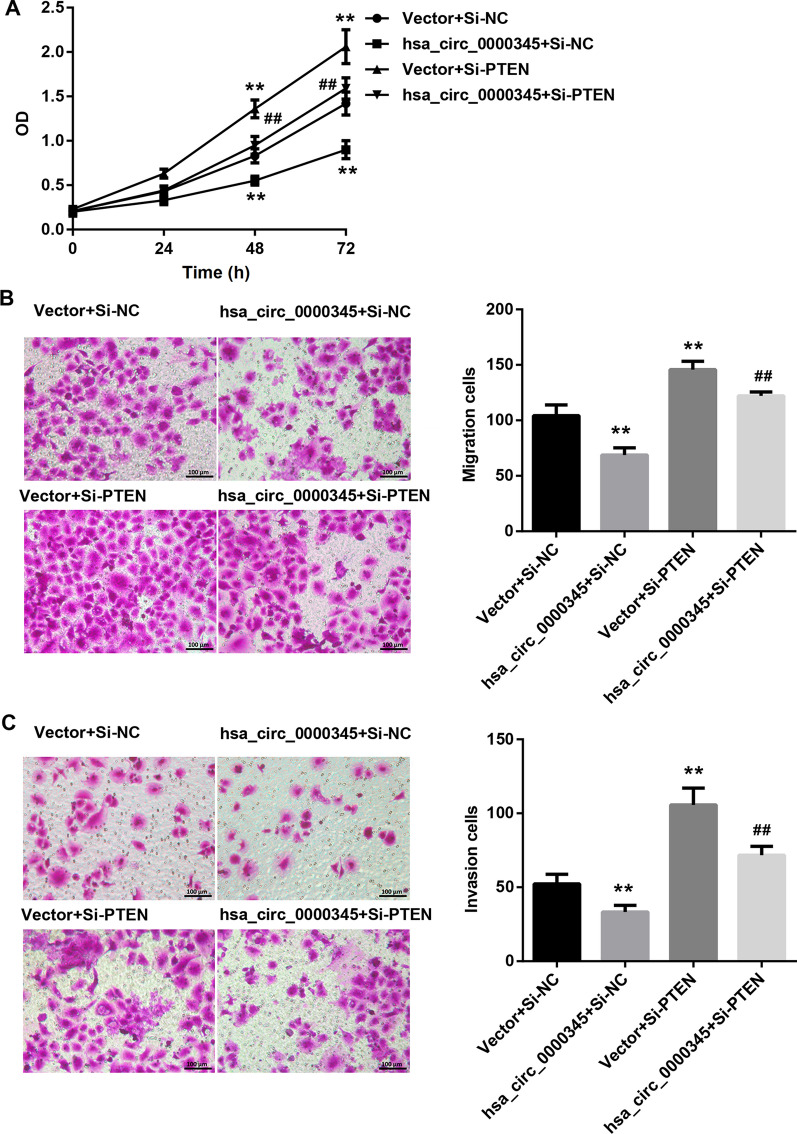
Hsa_circ_0000345 repressed cell proliferation, migration and invasion of SUNE1 cells by promoting PTEN. SUNE1 cells were co-transfected with pcDNA3.1-hsa_circ_0000345 or pcDNA3.1-NC and Si-PTEN or Si-NC. **A** MTT assay was performed to examine cell proliferation of the transfected SUNE1 cells. **B**, **C** Transwell assay was performed to estimate cell migration and invasion of the transfected SUNE1 cells. **P < 0.01 vs. Vector + Si-NC; ^##^*P* < 0.01 vs. Vector + Si-PTEN

Finally, we elucidated whether hsa_circ_0000345 can regulate cell proliferation, migration and invasion of NPC cells through miR-513a-3p/PTEN axis. As shown in Fig. 4A–C, hsa_circ_0000345 overexpression caused a decrease in cell proliferation, migration and invasion of SUNE1 cells. However, miR-513a-3p overexpression significantly enhanced cell proliferation, migration and invasion of SUNE1 cells. The influence conferred by miR-513a-3p overexpression was partly abolished by hsa_circ_0000345 up-regulation (Fig. 4A–C). Also, cell proliferation, migration and invasion were significantly reduced in SUNE1 cells in the presence of pcDNA3.1-hsa_circ_0000345. In contrast, PTEN knockdown led to an increase in cell proliferation, migration and invasion of SUNE1 cells. Hsa_circ_0000345 up-regulation impaired PTEN silencing-mediated promotion of cell proliferation, migration and invasion of SUNE1 cells (Fig. 5A–C). Taken together, hsa_circ_0000345 repressed cell proliferation, migration and invasion of NPC cells by regulating miR-513a-3p/PTEN axis.

## Discussion

CircRNAs are closely related to the occurrence and development of various diseases, especially cancer. For instance, Tan et al. have confirmed that hsa_circ_0001730 exerts antitumor effects in hepatocellular carcinoma by inhibiting cell proliferation, migration and invasion of hepatocellular carcinoma cells, which attributes to inhibit HIF-1α [16]. Circ_0000215 is highly expressed in tumor tissues of NPC, and circ_0000215 contributes to malignant phenotypes of NPC through regulating miR-512-5p/PIK3R1 axis [17]. Hsa_circ_0000345 has been reported to be associated with various diseases, such as atherosclerosis and radiation-induced liver disease. Hsa_circ_0000345 promotes cell viability and invasion and inhibits apoptosis of oxidized low density lipoprotein (ox-LDL)-treated aortic smooth muscle cells by promoting HIF-1α expression [18]. Hsa_circ_0000345 enhances CCND2 expression by competitive binding miR-758, and then promotes cell growth, migration and tube formation of ox-LDL-treated human aortic endothelial cells [19]. Hsa_circ_0000345 elevates fibrotic phenotypes and inflammatory response of irradiated hepatic stellate cells through miR-146a-5p/RAC1 axis, thereby accelerating the development of radiation-induced liver disease [20]. Additionally, hsa_circ_0000345 has been reported to be abnormally expressed in NPC tissues and cells, suggesting that hsa_circ_0000345 may be associated with the progression of NPC [7]. In the present study, we first determined the biological role of hsa_circ_0000345 in NPC. Consistently, hsa_circ_0000345 was significantly down-regulated in NPC cells lines. Furthermore, hsa_circ_0000345 had an inhibiting effect on cell proliferation, migration and invasion of NPC cells. Thus, we speculated that hsa_circ_0000345 may function as a tumor suppressor in NPC.

MiR-513a-3p has a vital role in various tumors. Previous study has revealed that miR-513a-3p plays an important role in controlling migration of lung tumor cells by targeting ITG-β8 [21]. In ovarian cancer cells, the expression of miR-513a-3p is significantly decreased, miR-513a-3p inhibits epithelial–mesenchymal transition and promotes sensitivity of ovarian cancer cells to cisplatin by targeting HOXB7 [22]. The study of Qin el al. has confirmed that miR-513a-3p is a target of LINC00473, and LINC00473 deficiency promotes radiosensitivity of non-small cell lung cancer cells via sponging miR-513a-3p [23]. In this work, we first found that there were binding sites between hsa_circ_0000345 and miR-513a-3p, and miR-513a-3p interacted with PTEN. Hsa_circ_0000345 functioned as a competing endogenous RNA (ceRNA) to repress miR-513a-3p, which controlled its down-stream target PTEN. Thus, these data suggested that hsa_circ_0000345 participated in the progression of NPC through regulating miR-513a-3p/PTEN axis.

PTEN as a tumor suppressor gene, participates in the progression of NPC. PTEN functions as a target of PAG1, and is repressed by PAG1 in NPC tissues and cells. PAG1 enhances cell proliferation and metastasis of NPC cells by interacting with PTEN [13]. Long non-coding RNA IUR promotes PTEN expression by competitive binding miR-144, and IUR/miR-144/PTEN axis suppresses proliferation of tumor cells in NPC [15]. MiR-182 accelerates cell proliferation and invasion of NPC cells by interacting with PTEN, thereby promoting the progression of NPC [24]. Consistently, we also confirmed the anticancer effect of PTEN in NPC. Our data demonstrated that hsa_circ_0000345 repressed cell proliferation, migration and invasion of NPC cells through miR-513a-3p/PTEN axis, thus aggravating the malignant progression of NPC.

## Conclusions

In conclusion, our study first demonstrates the biological role of hsa_circ_0000345 in NPC. Hsa_circ_0000345 inhibits cell proliferation, migration and invasion of NPC cells via miR-513a-3p/PTEN axis, thereby suppressing the progression of NPC. Thus, this work highlights a novel ceRNA circuitry involving key regulators of NPC, which may become a potential therapeutic target for NPC.

## Data Availability

The datasets used and/or analyzed during the current study are available from the corresponding author on reasonable request.
